# Dietary Bile Acids Supplementation on Growth Performance and Metabolism in Pacific White Shrimp (*Litopenaeus vannamei*)

**DOI:** 10.1155/anu/1329200

**Published:** 2025-11-10

**Authors:** Lei Zhang, Jinzhi Zhang, Peng Tan, Jun Zhang, Aijun Zhu, Zhili Ding, Xiaojun Yan, Qingjun Shao

**Affiliations:** ^1^School of Marine Sciences, Ningbo University, Ningbo 315211, China; ^2^Key Laboratory of Mariculture and Enhancement, Zhejiang Marine Fisheries Research Institute, Zhoushan 316021, China; ^3^College of Animal Sciences, Zhejiang University, Hangzhou 310058, China; ^4^Dezhou Bile Acids R&D and Application Key Laboratory, Bile Acids Animal Health Dezhou Engineering Research Center, Dezhou 253000, China; ^5^Zhejiang Provincial Key Laboratory of Aquatic Resources Conservation and Development, College of Life Science, Huzhou University, Huzhou 313000, China; ^6^National Engineering Research Center of Marine Facilities Aquaculture, Zhejiang Ocean University, Zhoushan 316022, China

**Keywords:** bile acids, growth performance, lipid metabolism, *Litopenaeus vannamei*, metabolomics

## Abstract

Optimizing bile acids (BAs) inclusion in commercial diets and understanding its molecular mechanisms in Pacific white shrimp (*Litopenaeus vannamei*) are crucial for improving growth performance. The present study investigated the effect of dietary BAs on shrimp growth and metabolic mechanisms using multiomics approaches. A total of 1800 shrimp (0.16 ± 0.01 g) were fed six diets with BAs levels: 0 mg kg^−1^ (control, BA0), 50 mg kg^−1^ (BA1), 100 mg kg^−1^ (BA2), 150 mg kg^−1^ (BA3), 200 mg kg^−1^ (BA4), and 250 mg kg^−1^ (BA5). After 56 days, the growth performance of BA4 and BA5 groups was significantly higher than the BA0, BA1, and BA2 groups. Regression analysis indicated an optimal dietary BAs level of 216−218 mg kg^−1^. Biochemical analysis showed that the BA4 group significantly reduced crude lipid content, triglycerides, and nonesterified fatty acids, while increasing digestive enzyme activities. RNA-seq revealed reduced expression of lipid synthesis genes (*srebp1*, *fasn*, *scd*, etc.), while enhancing the expression of genes related to lipid catabolism (*acox1*, *angpt4*, *dip13a*, and *gba*) and digestive enzymes (*prss1* and *prss2*). Metabolomic analysis showed taurine-conjugated BAs as predominant, with taurine content decreasing likely due to its conjugation with BAs and loss in feces. These results suggest that the optimal BAs level of 216−218 mg kg^−1^ improved shrimp growth and lipid metabolism by enhancing nutrient digestion, promoting lipolysis, and inhibiting lipid synthesis, without altering the BAs pool balance. This study first revealed taurine's critical role in Pacific white shrimp BAs metabolism.

## 1. Introduction

The rapid growth of the aquaculture industry has brought challenges associated with high-density and intensive farming practices, often leading to growth retardation, metabolic disruptions, and impaired immune function in various aquatic species [1]. To overcome these obstacles, functional feed additives have been incorporated into aquaculture diets to improve growth performance and metabolism [2–6]. Bile acids (BAs) have become prominent among feed additives due to their growth-promoting properties and are now widely utilized in diverse marine species. The positive impacts of BAs are particularly noted in low fish meal diets [7, 8], plant-based diets [9, 10], and energy-rich diets [4, 11], where they have been well-documented to enhance fish growth performance and metabolic disorders.

BAs are essential to regulating metabolism, particularly by improving the emulsification, digestion, and uptake of dietary fats. By the amphipathic structure, BAs solubilize lipids by forming micelles, thereby increasing the efficiency of nutrient utilization (e.g., lipids, cholesterol, and lipid-soluble vitamins) and promoting growth [12–15]. BAs also participate in enterohepatic circulation, this process is essential for nutrient uptake and allocation, modulates metabolic processes, and maintains energy balance within the body [16–20]. Additionally, BAs regulate the metabolism of aquatic animals by interacting with other nutrients such as taurine [6, 21–23], cholesterol [5], and lipids [4, 24]. Taurine is the main amino acid conjugating with BAs in most teleost fish. Studies have shown that dietary taurine supplementation can increase the synthesis of taurine-conjugated BAs, promote BAs and cholesterol metabolism, and improve growth performance [21–23]. The nutritional efficacy of BAs may be closely dependent on taurine availability, and insufficient taurine supply could limit BA conjugation efficiency and attenuate their physiological effects.

However, the influence of BAs on aquatic species' growth development and metabolism demonstrates notable heterogeneity, mediated by BA concentration gradients, species, and formulation of basal diets [10, 25]. The optimal concentration of BAs in Pacific white shrimp (*Litopenaeus vannamei*) feed varies significantly and is highly dependent on the composition of the basal diet. Su et al. [26] reported that adding 200−300 mg kg^−1^ of BAs to Pacific white shrimp feed significantly enhanced growth performance, lipid metabolism, and antioxidant capacity. Notably, the dietary fish meal inclusion level was unusually high at 270 g kg^−1^ in their study, whereas typical commercial Pacific white shrimp feeds contain only 100−250 g kg^−1^ fish meal [27]. Subsequently, Wang et al. [28] developed a commercial diet with 140 g kg^−1^ fish meal and experimented with different BAs levels (75, 150, and 300 mg kg^−1^). According to their study, Pacific white shrimp's growth, feed utilization, and physiological indicators were all enhanced by 150 mg kg^−1^ of BAs, although it did not establish an optimal BAs inclusion level. Additionally, Li et al. [29] investigated the effects of gradient BAs levels (150, 300, 600, and 900 mg kg^−1^) in 100 g kg^−1^ fish meal diets. Their results indicated that up to 900 mg kg^−1^ could significantly enhance the shrimp growth performance. These studies underscore the potential of BAs to enhance growth performance and metabolic efficiency in Pacific white shrimp diets, while also stressing the importance of precisely determining BAs levels tailored to specific feed formulations. To maximize growth benefits while minimizing potential adverse effects, it is essential to determine the optimal level of BAs in commercial feeds with a moderate level of fish meal inclusion.

Research into the effects of BAs supplementation on the growth and metabolism of aquatic invertebrates is scientifically significant, especially given that invertebrates cannot synthesize BAs internally [3, 30]. Given the substantial differences in BAs composition and metabolism between fish and invertebrates, it is crucial to investigate whether these species share similar metabolic mechanisms when supplemented with dietary BAs. Recent studies have shown that BAs prompt the growth of invertebrate species such as hybrid abalone (*Haliotis discus hannai*) [24, 31], Chinese mitten crab (*Eriocheir sinensis*) [12], and giant freshwater prawn (*Macrobrachium rosenbergii*) [32]. Furthermore, supplementing with BAs improved intestinal and hepatopancreatic health, while also increasing growth performance, lipid metabolism, immunological response, and antioxidant capacity in Pacific white shrimp [5, 8, 26, 28, 29] and black tiger shrimp (*Penaeus monodon*) [33]. However, the specific molecular mechanisms through which dietary BAs levels affect growth and metabolism in Pacific white shrimp remain unclear. Omics technologies offer a robust and comprehensive approach to sample analysis that extends beyond single metrics. The integration of transcriptomes and metabolomics provides a deeper understanding of the relationships between key genes and metabolites, enabling the exploration of underlying biological mechanisms [34].

The present study aims to investigate the effects of BAs on the growth and metabolism of Pacific white shrimp and determine the optimal concentration of BAs in commercial feeds. Six experimental diets were formulated, each containing 220 g kg^−1^ fish meal, with BAs concentrations ranging from 0 to 250 mg kg^−1^. A 56-day feeding and growth experiment was done to identify the optimal BAs concentration. Additionally, transcriptomic and metabolomic methods were employed to explore the impact of BAs on metabolic genes and key metabolic substances. The findings enhance understanding of the metabolic mechanisms of BAs in invertebrates and may provide insights into the development of feed additives for *Litopenaeus vannamei*.

## 2. Materials and Methods

**Figure 1:**
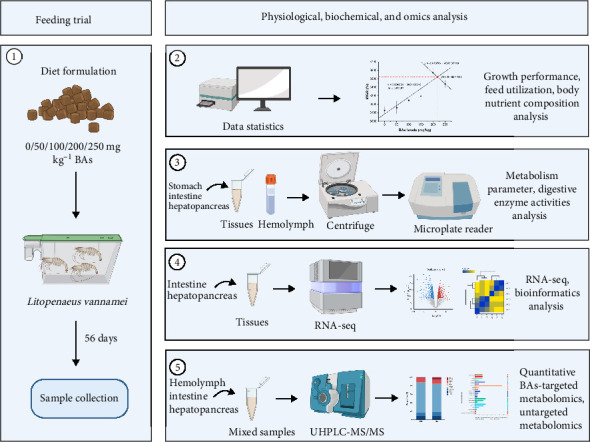
The overall workflow of the study. Pacific white shrimp (0.16 ± 0.01 g) were fed diets with varying BAs levels (0, 50, 100, 150, 200, and 250 mg kg^−1^) over a 56-day feeding trial. Key assessments include growth performance, feed utilization, shrimp body nutrient composition, metabolism parameters, and digestive enzyme activities. Omics analyses, including RNA sequencing and metabolomics, were employed to explore the molecular mechanisms driving the observed outcomes.

The Ethics Board on Animal Experiments approved the investigation at the Zhejiang Marine Fisheries Research Institute and was conducted in full adherence to ethical standards and guidelines for Aquaculture Nutrition.  Figure 1 shows the general workflow and provides an overview of the present study.

### 2.1. Diet Preparation and Pacific White Shrimp Husbandry

The proximate composition of feed components, trial diets, and shrimp specimens were quantified using the Association of Official Analytical Chemists (AOAC) methods [35]. The primary protein sources were fish meal, soybean meal, and soy protein concentrate, while fish oil and soybean oil served as the main lipid sources. Detailed nutrient compositions are presented in Table 1. High-purity (99%) BAs were obtained from Shandong Longchang Animal Health Product Co., Ltd., China, and incorporated into the diets. Six experimental diets were formulated, each containing 400 g kg^−1^ crude protein, 80 g kg^−1^ crude lipid, and a total energy content of 16.33 MJ kg^−1^. BAs were supplemented at 0, 50, 100, 150, 200, and 250 mg kg^−1^, with corresponding diet groups labeled BA0 to BA5, as detailed in Table 1. Feed pellets (1.20 mm diameter) were steam-sterilized according to the procedure outlined by Xu et al. [36].

Healthy Pacific white shrimp from the same spawn were acclimated for 2 weeks on a commercial diet (52% protein, 8% lipid, 10% moisture, and 17% ash). Subsequently, 1800 juveniles (0.16 ± 0.01 g) were randomly distributed into 36 tanks (50 shrimp per tank) within a flowing water system (1 L min^−1^). Each 500 L opaque blue polypropylene aquaculture tank, containing 400 L of seawater filtered through a sand filter, with six replicates per diet treatment. Over 56 days, the water temperature, pH, nitrite, unionized ammonia, and dissolved oxygen were maintained at 25.0 ± 1.5°C, 7.7 ± 0.1, <0.1 mg L^−1^, <0.05 mg L^−1^ and >6.0 mg L^−1^, respectively. Specified test diets were given to the shrimp three times a day, at 6:00, 12:00, and 18:00. Feces and leftover feed were removed 3 h following each feeding session to keep the tank clean and stop the deterioration of the water quality.

### 2.2. Sample Collections

Prior to the experiment, about 1000 juveniles were randomly chosen for preliminary body composition analysis and stored at −20°C. After a 56-day feeding trial, shrimp were fasted for 24 h to standardize metabolic conditions. They were then immobilized on an ice plate to minimize stress and weighed individually after remaining stationary.

For the purpose of analyzing the whole-body proximate composition, 10 juveniles were randomly selected from each aquarium. For hemolymph biochemical assessments, hemolymph was drawn from the hearts of the remaining shrimp, and centrifuged to obtain the supernatant before storage at −80°C. The hepatopancreas, stomach, and intestines were carefully dissected and weighed during ice-based visceral mass extraction to assess digestive enzyme activity.

A total of five juveniles per tank were randomly collected, and their hepatopancreas and intestines were stored in an RNA storage solution (Cowin Biotech, Beijing, China) at −20°C for RNA sequencing analysis. Another set of five juveniles per tank was randomly sampled, with a mixed hepatopancreas, intestine, and hemolymph sample stored at −80°C for metabolomics analysis.

### 2.3. Biochemical Analysis

In accordance with our earlier reports, the stomach, hepatopancreas, and intestinal tissues were pretreated [37]. The resulting supernatants were then preserved at −80°C for future assessment of digestive enzymes. Hemolymph samples were analyzed for biochemical indicators. The manufacturer's guidelines were strictly adhered to during assay procedures with commercially available kits.

### 2.4. RNA Sequencing and Bioinformatics Analysis

Total RNA was extracted from the intestine and hepatopancreas tissues, and RNA integrity, purity, and concentration were assessed following the protocol of Zhang et al. [38]. After initial sample preparation, RNA samples were sent for testing. Quality control, RNA sequencing, and differentially expressed gene (DEG) identification followed our previously described methods [38]. Significant DEGs were identified based on a corrected *p*-value <0.05 and a Log_2_-fold change >1. Postsequencing, pathway analyses were carried out to investigate the biological functions and connections of the identified genes.

### 2.5. Gene Expression Analysis

Tissue RNA extraction, quantification, and quality assessment followed Zhu et al. [39]. To ensure that RNA samples were free of genomic DNA, RNase-free DNase (Takara Bio Inc., Japan) was used during RNA processing. Using sequence-specific primers (Table S1), qRT-PCR was conducted following the methodology detailed in our earlier research [40]. Gene expression quantification was determined using the 2^−*ΔΔ*CT^ method, normalized against the geometric mean of two reference genes for enhanced accuracy and consistency [41].

### 2.6. Metabolomics Analysis

#### 2.6.1. Quantitative BAs-Targeted Metabolomics

The samples underwent homogenization in 500 μL of an acetonitrile/methanol mixture (8:2, *v*/*v*) followed by centrifugation (12,000 rpm and 20 min) for protein depletion. The clarified supernatant underwent gentle nitrogen-mediated evaporation, reconstitution in 100 μL of acidified aqueous solution (0.1% formic acid in water/acetonitrile, 2:8, *v*/*v*), and injection of 2 μL for UHPLC-MS/MS analysis. Analyses were performed on an ExionLC AD UHPLC-QTRAP 6500+ system (AB SCIEX Corp., Boston, MA, USA) using a Waters ACQUITY UPLC BEH C18 column. The mobile phase consisted of 0.1% formic acid in water (solvent A) and acetonitrile (solvent B), with a gradient elution from 20% to 100% B over 12.5 min, held for 2 min, and returned to 20% B, completing a 17 min run at 50°C and 0.30 mL min^−1^. The injection volume was 10 μL.

#### 2.6.2. Untargeted Metabolomics

Approximately 100 mg of tissue samples were pulverized under liquid nitrogen and resuspended in prechilled 80% methanol. After vortexing and incubating on ice for 5 min, the mixture was centrifuged at 15,000 × *g*, 4°C, for 20 min. The supernatant was diluted to 53% methanol, centrifuged again, and prepared for LC-MS/MS analysis. Metabolomic profiling was conducted using a Vanquish UHPLC system coupled with an Orbitrap Q Exactive HF or HF-X mass spectrometer (Thermo Fisher Scientific, Bremen, Germany). Principal component analysis (PCA) and partial least squares discriminant analysis (PLS-DA) were applied to identify data patterns and discriminant metabolites. Significant metabolites were defined by VIP scores >1, *p*-values <0.05, and fold changes beyond ±1.

### 2.7. Data Computation and Statistical Analyses

The subsequent parameters were calculated:

 
Survival rate (SR, %)=100 × *N*_*t*_/*N*_0_ [8];
 
Weight gain rate (WGR, %)=100 × (*W*_*t*_ − *W*_0_)/*W*_0_ [40];
 
Specific growth rate (SGR, %day^−1^)=100 × (Ln*W*_*t*_– Ln*W*_0_)/*t* [8];
 
Condition factor (CF, %)=100 × (Final body weight)/(final body length)^3^ [42];
 
Hepatosomatic index (HSI, %)=100 × (Hepatopancreas weight/*W*_*t*_) [5];
 
Feed intake (FI, %day^−1^)=100 × Dry feed intake/[(initial body weight+ final body weight)/2]/*t* [42];
 
Feed conversion ratio (FCR)=Dry feed fed/wet weight gained [5, 26];
 
Protein efficiency ratio (PER)=(*W*_*t*_ − *W*_0_)/protein intake [28];
 
Protein productive value (PPV, %)=100 × Protein gain/total protein intake [36].

In this study, *W*_*t*_ and *W*_0_ represent the total final and initial body weights of the Pacific white shrimp, respectively; *N*_*t*_ and *N*_0_ signify the final and initial number of Pacific white shrimp in each container, correspondingly, while *t* represents the duration of the experiment in days.

Statistical analyses were conducted using SPSS 20.0 (SPSS Inc., Chicago, IL, USA), with results reported as mean ± standard error of the mean (S.E.M.). Homogeneity of variance and data normality were assessed via Levene's test. For nonnormally distributed data, the Kruskal–Wallis test was employed, whereas one-way ANOVA followed by Tukey's multiple range test was utilized for normally distributed data. The significance of the regression models, including linear, quadratic, and cubic regression models, was evaluated using the orthogonal polynomial comparison method [43]. Regression models were applied utilizing Origin 2021 software (OriginLab Corporation, Northampton, MA, USA).

## 3. Results

### 3.1. Effects of Dietary BAs Levels on Growth Performance, Feed Utilization, and Shrimp Body Nutrient Composition

**Figure 2:**
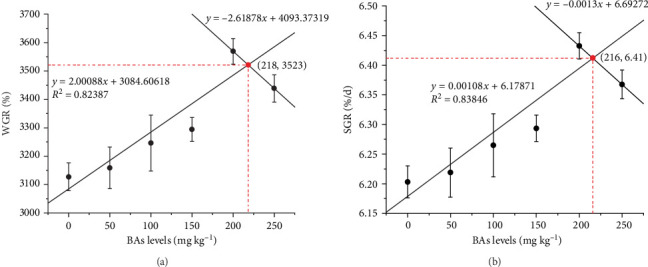
Assessing the optimal BAs levels by linear regression analysis. WGR (A) and SGR (B) of Pacific white shrimp fed BAs gradients for 56 days were analyzed using regression. BAs, bile acids; SGR, specific growth rate; WGR, weight gain rate.

Table 2 data indicate no significant influence in SR, CF, HSI, FI, FCR, PER, or PPV following BA supplementation (*p* > 0.05). However, FBW, WGR, and SGR improved with increasing BAs levels, peaking at 200 mg kg^−1^ (BA4; *p* < 0.05). Further increases to 250 mg kg^−1^ provided no additional benefits but remained superior to the control (*p* < 0.05). Regression analysis identified 216–218 mg kg^−1^ as the optimal dietary BAs level for growth performance (Figure 2). Regarding shrimp nutritional composition, dietary BAs supplementation did not markedly alter moisture, crude protein, or ash content (*p* > 0.05), but crude lipid content decreased significantly with increasing BAs levels compared to the BA0 group (*p* < 0.05).

### 3.2. Effects of Dietary BAs Levels on Hemolymph Metabolism Parameters and Digestive Enzyme Activities

As shown in Table 3, dietary BAs at 150 mg kg^−1^ (BA3), 200 mg kg^−1^ (BA4), and 250 mg kg^−1^ (BA5) significantly reduced TG content compared to BA0, BA1, and BA2 (*p* < 0.05). Additionally, 200 mg kg^−1^ (BA4) significantly lowered NEFAs content relative to all other groups (*p* < 0.05). However, GLU, TC, HDL-c, and LDL-c levels did not significantly change across treatments (*p* > 0.05).

Table 4 indicated that stomach trypsin, lipase, and amylase activities were not significantly affected by dietary BAs supplementation (*p* > 0.05), as did intestinal protease and amylase, along with hepatopancreatic amylase activity (*p* > 0.05). However, hepatopancreatic trypsin activity increased with higher BAs levels. Lipase activity in the hepatopancreas was significantly enhanced in the BA2–BA5 groups (100–250 mg kg^−1^) compared to BA0 and BA1 (*p* < 0.05). Similarly, intestinal lipase activity was substantially higher among the BA3–BA5 groups (150–250 mg kg^−1^) than that in BA0–BA2 (*p* < 0.05).

### 3.3. RNA Sequencing Analysis and DEG Enrichment

RNA sequencing identified 24,680 annotated genes, with clean reads aligned to the reference genome (GCF_003789085.1) at rates of 76.13%–87.86% using BLAST. Functional annotation covered 63.82% of genes in Gene Ontology (GO), 56.14% in Kyoto Encyclopedia of Genes and Genomes (KEGG), and 97.26% in the nonredundant protein sequence database (NR), producing an overall annotation rate of 97.38%.

**Figure 3:**
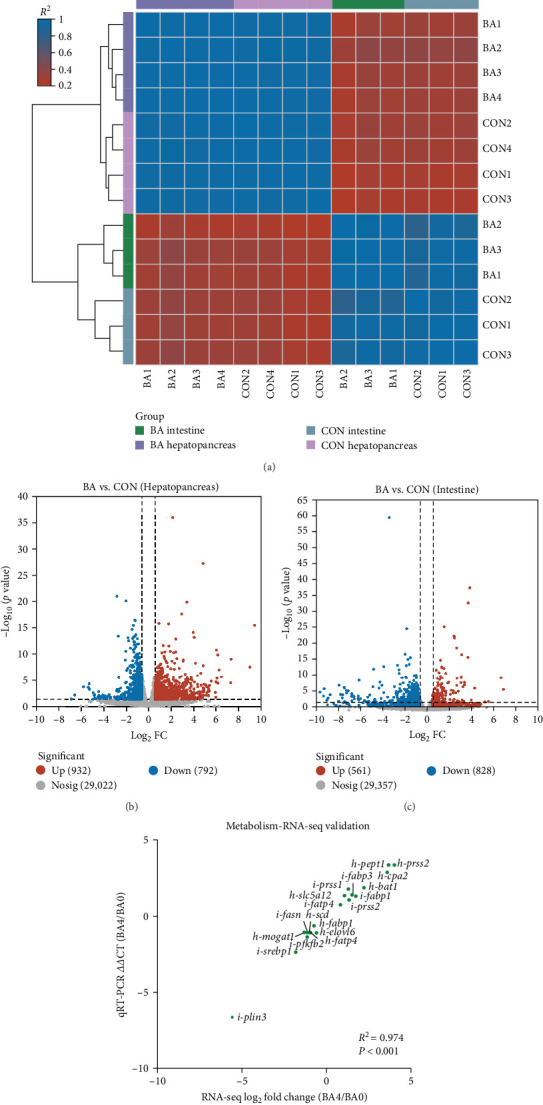
RNA-seq analysis of shrimp hepatopancreas and intestinal tissues. (A) Pearson correlation between hepatopancreas and intestinal tissue samples. (B, C) Volcano plots comparing gene expression levels in the hepatopancreatic and intestinal tissues of the BA and CON groups, respectively. (D) Verification of RNA-seq data through qRT-PCR analysis of selected DEGs.

Pearson correlation analysis revealed strong intragroup consistency as well as significant between-group variability between BA0 (CON) and BA4 (BA) groups (Figure 3A). DESeq2 differential expression analysis identified 932 upregulated and 792 downregulated genes in the hepatopancreas (Figure 3B), and 561 upregulated and 828 downregulated genes in the intestine (Figure 3C), all with Log_2_-fold change >1 and adjusted *p* < 0.05. qRT-PCR measured the mRNA expression levels of 18 chosen genes to confirm RNA-seq data validity, demonstrating the same trend as the transcriptome analysis with a correlation coefficient of 0.974 (*p* < 0.001; Figure 3D).

**Figure 4:**
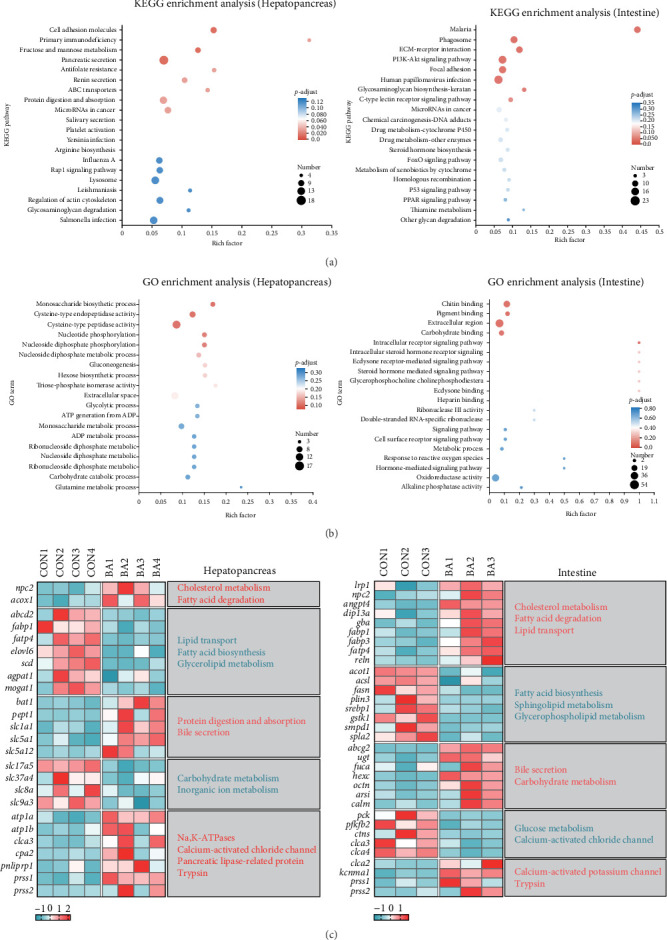
DEGs enrichment analysis and heatmap analysis. (A) KEGG enrichment analysis. (B) GO enrichment analysis. (C) Heatmap of DEGs selected from hepatopancreas and intestinal tissues.

KEGG and GO enrichment analyses highlighted key pathways for DEGs (Figure 4A,B). In the hepatopancreas, the most enriched KEGG pathway was cell adhesion molecules, alongside fructose and mannose metabolism, pancreatic secretion, and protein digestion and absorption. GO enrichment emphasized glycometabolism and nucleotide metabolism. In the intestine, the top KEGG pathway was malaria, followed by peroxisome proliferator-activated receptor (PPAR) signaling, forkhead box O (FoxO) signaling, and steroid hormone biosynthesis. GO analysis highlighted lipid metabolism and oxidoreductase activity as key enriched categories.

DEG expression trends in the hepatopancreas and intestine were depicted in the heatmap (Figure 4C). It revealed a significant upregulation of genes related to cholesterol metabolism (*npc2* and *lrp1*), fatty acid degradation (*acox1*, *angpt4*, *dip13a*, and *gba*), BA secretion (*slc5a1*, *abcg2*, and *ugt*), and trypsin (*prss1* and *prss2*) in both the hepatopancreas and intestine. Additionally, the expression of hepatopancreatic pancreatic lipase-related protein (*pnliprp1*) was markedly increased. Conversely, the heatmap showed significant downregulation of genes involved in lipid transport (*fabp1* and *fatp4*), fatty acid synthesis (*elovl6* and *scd*), and triglyceride metabolism (*agpat1* and *mogat1*) in the hepatopancreas. In the intestinal tissue, genes associated with fatty acid biosynthesis (*acot1*, *acsl*, *fasn*, *plin3*, *srebp1*, and *gstk1*), glycerophospholipid metabolism (*spla2*), and glucose metabolism (*pck* and *pfkfb2*) were also significantly downregulated.

### 3.4. BAs-Targeted Metabolomics

**Figure 5:**
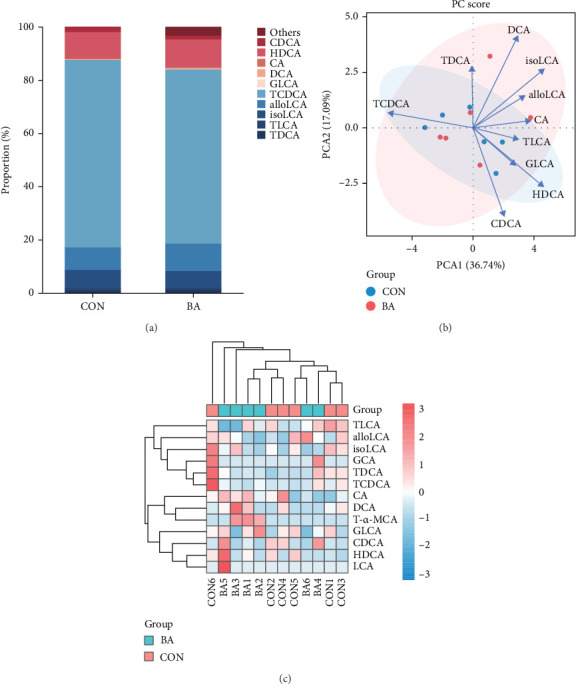
Composition and correlation analysis of BAs in the Pacific white shrimp. (A) The proportion of several major BAs in the total pool. alloLCA, allolithocholic acid; CA, cholic acid; CDCA, chenodeoxycholic acid; DCA, deoxycholic acid; GLCA, glycolithocholic acid; HDCA, hyodeoxycholic acid; isoLCA, isolithocholic acid; TCDCA, taurochenodeoxycholic acid sodium salt; TDCA, taurodeoxycholic acid sodium salt; TLCA, taurolithocholic acid sodium salt. Others: it includes lithocholic acid (LCA), tauro-α-muricholic acid sodium salt (T-α-MCA), and glycocholic acid hydrate (GCA), which were not detected or below detection limit. (B) For each of the 13 BAs, the BA profiles were subjected to principal component analysis (PCA). Oval circles represent the 95% confidence intervals. The original variables are represented by arrows, and each original variable's contribution to the principal component is shown by projecting the arrows onto the coordinate axes. The correlation between the principal component and the original variables is shown by the arrows' direction. (C) Heatmap showing BAs profile clustering analysis. The chromatic gradient reflects the expression level of BAs (red, high expression; blue, low expression).

The BAs profiles in shrimp were analyzed using UHPLC-MS/MS, which separated 25 BAs/BAs salt standards within a 16-min run, including column equilibration (Table S2). The method met the required standards for matrix effects, precision, accuracy, and stability. Among the 25 analyzed BAs, 13 were detected, while the remaining 12 were below quantification limits (Figure 5A and Table S2). The most abundant BAs were TCDCA, HDCA, alloLCA, isoLCA, and TDCA. BAs composition showed negligible differences between the BA0 (CON) and BA4 (BA) groups. PCA analysis revealed no clear separation between groups (Figure 5B), and the heatmap displayed similar trends (Figure 5C), suggesting that the optimal levels of dietary BAs supplementation did not significantly alter the BAs profiles in Pacific white shrimp.

### 3.5. Untargeted Metabolomics

**Figure 6:**
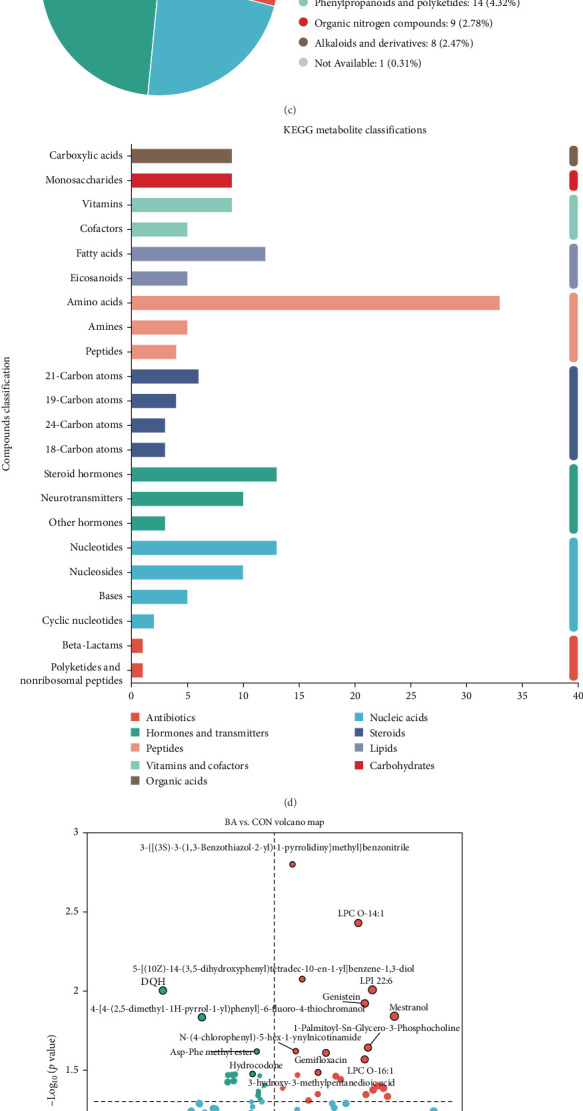
Nontarget metabolomic analysis of Pacific white shrimp hepatopancreas samples. (A) The score plot of the PLS-DA model between the BA and CON groups. (B) The plot of the PLS-DA permutation test between the BA and CON groups. (C) HMDB metabolite classifications. (D) KEGG metabolite classifications. (E) Volcanic map of differential metabolites. (F) KEGG pathway enrichment abundance score map of differential metabolites.

The PLS-DA score plot demonstrated strong intragroup clustering and clear separation between groups, indicating a significant impact of BAs on hepatopancreatic metabolism in Pacific white shrimp (Figure 6A). The PLS-DA permutation test confirmed model robustness, with a *Q*^2^ regression line intercept below 0.05, ensuring reliability and absence of overfitting (Figure 6B). Metabolite annotation was performed using the HMDB and KEGG databases. In HMDB, the top three metabolite categories were lipids and lipid-like molecules, organic acids and derivatives, and organoheterocyclic compounds (Figure 6C). In KEGG, the most represented categories were amino acids, fatty acids, and steroid hormones (Figure 6D). In the BA group, 22 metabolites were upregulated, while 15 were downregulated in contrast with the CON group (Figure 6E). The KEGG enrichment analysis highlighted the significant involvement of pathways related to taurine and hypotaurine metabolism, alongside sulfur metabolism (Figure 6F). Notably, a significant reduction in taurine levels was observed within the BA group relative to the CON group.

## 4. Discussion

The optimal level of exogenous BAs significantly improves growth performance in aquatic species. A study on grass carp (*Ctenopharyngodon idella*) indicated that 160 mg kg^−1^ of BAs supplementation enhanced growth performance, while dietary 320 mg kg^−1^ BAs supplementation lead to retard growth [44]. Dietary optimal levels of BAs in other fish species prominently increased their growth performance; however, the optimal level varied by species: 150 mg kg^−1^ in tilapia (O*reochromis niloticus*) [2], 300 mg kg^−1^ in large yellow croaker (*Larimichthys crocea*) [11], and 475 mg kg^−1^ in largemouth bass (*Micropterus salmoides*) [45]. Studies on fish species have concluded that an appropriate level of dietary BAs can promote growth, while excessive BAs supplementation exerts cytotoxic effects, thereby inhibiting fish growth [25]. Distinct from fish species, Pacific white shrimp cannot synthesize BAs endogenously. Previous researches have shown that dietary optimal BAs supplementation can improve Pacific white shrimp growth performance [5, 8, 26, 33]. A similar result was obtained in the present study, and the optimal BAs level was estimated at 216–218 mg kg^−1^ based on the growth parameters.

The growth-promoting effects of exogenous BAs could be linked to enhanced feed utilization. Studies on large yellow croaker have shown that exogenous BAs supplementation significantly increased PER and reduced FCR, leading to improved WGR and SGR [11]. The positive impacts of BAs were also been evidenced in European eel (*Anguilla anguilla*), where BAs significantly improved protein utilization efficiency [46]. Similarly, Wang et al. [28] reported that exogenous supplementation of 150 mg kg^−1^ BAs significantly reduced the FCR and increased growth performance in Pacific white shrimp. In the present study, a decreasing trend of FCR and increasing PER and PPV was noted with BAs supplementation.

The exogenous BAs had a significant impact on lipid metabolism, mainly by enhancing lipid digestion and absorption, as well as promoting lipid catabolism. BAs can emulsify lipids to form small micelles, increasing the surface area for lipase–lipid interactions and promoting the hydrolysis of ester bonds [10, 47], which enhances the digestion and absorption of lipid. Additionally, BAs activate bile salt-activated lipase [48] and enhance lipid utilization by forming water-soluble compounds with glycerol fatty acids [49]. The present study revealed that an optimal level of exogenous BAs significantly increased hepatopancreatic and intestinal lipase activity, which was consistent with the findings of Su et al. [26] in Pacific white shrimp. Additionally, BAs can also activate lipase activity in other aquatic animals, such as large yellow croaker [11], tongue sole (*Cynoglossus semiliaevis*) [50], Japanese flounder (*Paralichthys olivaceus*) [51], and leopard coral grouper (*Plectropomus leopardus*) [52], thereby improving feed utilization. Dam et al. [53] reported that the digestion and absorption of nutrients are related to genes encoding digestive enzymes. In the present study, it was observed that an optimal level of BAs significantly upregulated the expression of digestive enzyme-encoding genes (such as *prss1*, *prss2*, and *pnliprp1*), indicating the potential beneficial effect of BAs on upregulating digestion.

Although BAs enhanced lipid digestion and absorption, it is noteworthy that dietary BAs supplementation did not lead to abnormal lipid metabolism but rather exhibited a promoting effect on lipid catabolism. In the current investigation, increasing dietary BAs supplementation led to a reduction in TG levels within the hemolymph of Pacific white shrimp. This finding aligns with earlier research by Su et al. [5] and Li et al. [8]. The emulsifying effect of BAs can enhance the transport efficiency of TG, while also improving the clearance capacity of chylomicrons from the blood or slowing their release into the bloodstream [54]. Excessive NEFAs can accumulate in the hepatopancreas, thereby affecting the health of Pacific white shrimp [55]. In the current investigation, dietary BAs levels significantly decreased hemolymph NEFAs levels. This could be explained as the emulsifying effect of the BAs that facilitates the transport of NEFAs to the cell surface and accelerates lipid breakdown [54]. Transcriptomic results provided additional proof for the beneficial effects of BAs on lipid metabolism. RNA-seq indicated that BAs significantly increased the expression of lipid catabolism-related genes (*acox1*, *angpt4*, *dip13a*, and *gba*), while decreasing the expression levels of lipid synthesis-related genes (*srebp1*, *fasn*, and *scd*). Transcription factor SREBP1 modulates transcriptional activity of genes involved in fatty acid biosynthesis, including *fasn* and *scd* [56]. The downregulation of *srebp1* increased the lipid catabolism-related gene expression, thereby promoting fatty acid oxidation and catabolism [57]. These results were accordant with previous findings of Ding et al. [11] and Du et al. [58], where the diet-based supplementation with BAs lowered the expression of lipid synthesis genes such as *srebp1*, *fasn*, and *scd*, while enhancing the expression of the lipid oxidation gene *ppara* in large yellow croaker. Dietary BAs could decrease body lipids content by promoting the digestion of lipids and the subsequent breakdown of lipids for energy. Studies have shown that dietary BAs can reduce whole-body lipid content in species such as tiger puffer (*Takifugu rubripes*) [4], tilapia [2], rice field eel (*Monopterus albus*) [59], and grass carp [60]. These results coherently revealed that BAs not only promoted the digestion of dietary lipids but also improved overall lipid utilization, which reduced body lipid accumulation.

High-fat and low-fish meal diets significantly affect metabolism, leading to metabolic disorders, which subsequently lead to retard growth performance [25, 61]. Exogenous BAs compensate for these metabolic imbalances by improving the BAs profile and increasing the BAs pool size [29, 62]. To explore the potential metabolic influences of exogenous BAs, a metabolomics analysis was carried out. Results indicated exogenous BAs supplementation at 200 mg kg^−1^ did not significantly affect BAs pool profiles. One possible explanation is that the basal diet formulated with moderate fish meal content will not impair the normal BAs profiles. In such a balanced state, most of the exogenous BAs were finally excreted without significantly altering endogenous BAs profiles. To be noticed, TCDCA was found to be the predominant BAs in the shrimp BAs pool, a finding consistent with previous studies on large yellow croaker [39] and tiger puffer [21]. These results indicated that dietary BAs may primarily form conjugated BAs, particularly through taurine conjugation, leading to increased excretion in this form. The observed reduction in taurine levels in non-targeted metabolomics further supports this hypothesis, indicating taurine plays a crucial role in BAs metabolism. Previous studies have shown that dietary taurine supplementation increases taurine-conjugated BAs levels in various fish species, including tiger puffer [21], Korean rockfish (*Sebastes schlegeli*) [23], orange-spotted grouper (*Epinephelus coioides*) [6], and Japanese flounder [22]. Moreover, Huang et al. [63] reported a synergistic effect of taurine and BAs supplementation, which enhanced growth performance and regulated lipid metabolism in Pacific white shrimp. These findings suggest that taurine availability may influence the efficiency of BAs utilization, thereby affecting the regulation of lipid metabolism. Thus, determining the optimal feed formulation to maximize the effectiveness of BAs requires considering the influence of taurine.

## 5. Conclusions

This research examined how supplementing Pacific white shrimp's diet with varying levels of BAs affects their growth and metabolic processes. The conclusions were as follows:

1. Under the commercial feed formulation with a fish meal content of 220 g kg^−1^, the optimal range for BAs inclusion in Pacific white shrimp was estimated as 216–218 mg kg^−1^.
2. BAs enhanced digestive enzyme activity, enhanced lipid digestion and absorption, promoted lipid catabolism, and inhibited lipid synthesis.
3. Exogenous BAs supplementation at 200 mg kg^−1^ did not significantly affect BAs pool profiles in Pacific white shrimp.
4. Taurine-conjugated BAs constituted the predominant BAs, and taurine plays a critical role in BAs metabolism.

## Figures and Tables

**Table 1:** The formulation of the experimental diet and its fundamental nutritional constituents (g kg^−1^, dry weight).

Ingredients	Experimental diets					
	BA0^a^	BA1^b^	BA2^c^	BA3^d^	BA4^e^	BA5^f^
Fish meal^g^	220	220	220	220	220	220
Soybean meal^h^	160	160	160	160	160	160
Soy protein concentrate^i^	120	120	120	120	120	120
Shrimp meal^j^	50	50	50	50	50	50
Chicken meal^k^	60	60	60	60	60	60
Wheat flour^l^	220	220	220	220	220	220
Chicken liver powder^m^	40	40	40	40	40	40
Soy lecithin	20	20	20	20	20	20
Fish oil	20	20	20	20	20	20
Soybean oil	10	10	10	10	10	10
Ca(H_2_PO_4_)_2_	20	20	20	20	20	20
Vc phosphate	2	2	2	2	2	2
L-lysine	5	5	5	5	5	5
CaHMB^n^	4	4	4	4	4	4
Vitamin premix^o^	3	3	3	3	3	3
Mineral premix^p^	5	5	5	5	5	5
Bile acids (mg kg^−1^)^q^	0	50	100	150	200	250
Zeolite	30	30	30	30	30	30
Antioxidant^r^	1	1	1	1	1	1
Mold inhibitor^s^	1	1	1	1	1	1
Microcrystalline cellulose	9	9	8.9	8.9	8.8	8.8
Total	1000	1000	1000	1000	1000	1000
Nutritional level (Mean values, g kg^−1^, dry matter)						
Crude protein	402.8	406.7	404.4	405.2	406.6	404.5
Crude lipid	80.8	81.4	81.0	81.8	81.5	80.9
Ash	102.1	98.8	103.4	94.6	102.8	99.4
Energy (MJ kg^−1^)	17.08	16.79	16.84	16.69	16.55	16.37

^a–f^Bile acids (0, 50, 100, 150, 200, and 250 mg kg^−1^) incorporated into the diet.

^g–m^Fish meal: crude protein, 616.9 g kg^−1^; crude lipid, 90.0 g kg^−1^. Soybean meal: crude protein, 531.2 g kg^−1^; crude lipid, 26.4 g kg^−1^. Soy protein concentrate: crude protein, 624.0 g kg^−1^; crude lipid, 0.3 g kg^−1^; shrimp meal: crude protein, 581.8 g kg^−1^; crude lipid, 34.2 g kg^−1^. Chicken meal: crude protein, 683.5 g kg^−1^; crude lipid, 123.4 g kg^−1^. Wheat flour: crude protein, 145.8 g kg^−1^; crude lipid, 7.7 g kg^−1^. Chicken liver powder: crude protein, 485.7 g kg^−1^; crude lipid, 123.4 g kg^−1^.

^n^CaHMB: Calcium 2-Hydroxy-4-(methylthio) butyrate, 70% purity, was obtained from Shandong Xinhe Cheng Amino Acid Co., Ltd. (Weifang, Shandong, China).

^o,p^Vitamin and mineral premix (mg kg^−1^ diet): the study by Xu et al. [36] goes into detail about the specific ratios.

^q^Bile acids: Hyodeoxycholic acid 76.50%, chenodeoxycholic acid 17.04%, and hyocholic acids 3.14%.

^r^Antioxidant: Tertiary butbashydroquinone (TBHQ).

^s^Mold inhibitor: Calcium propionate and fumaric acid have a weight ratio of 1:1.

**Table 2:** Effect of dietary bile acids levels on growth, feed utilization, and body nutrients content.

Parameters	BA0	BA1	BA2	BA3	BA4	BA5	*p*-Value^1^	Pr > *F*^2^		
								Ln	Qd	Cu
SR (%)	91.33 ± 1.69	91.00 ± 1.13	94.33 ± 2.09	94.00 ± 1.55	93.67 ± 1.41	93.00 ± 1.34	0.55	—	—	—
FBW (g)	5.16 ± 0.08^c^	5.22 ± 0.12^c^	5.35 ± 0.16^c^	5.43 ± 0.07^bc^	5.87 ± 0.07^a^	5.66 ± 0.08^ab^	<0.001	0.02	0.12	0.19
WGR (%)	3127 ± 48.2^c^	3159 ± 73.4^c^	3246 ± 98.3^c^	3294 ± 42.10^bc^	3570 ± 45.3^a^	3439 ± 47.9^ab^	<0.001	0.02	0.12	0.19
SGR (% day^−1^)	6.20 ± 0.03^c^	6.22 ± 0.04^c^	6.26 ± 0.05^c^	6.29 ± 0.02^bc^	6.43 ± 0.02^a^	6.37 ± 0.02^ab^	<0.001	0.02	0.12	0.17
CF (%)	0.89 ± 0.02	0.90 ± 0.01	0.91 ± 0.01	0.91 ± 0.01	0.92 ± 0.01	0.93 ± 0.01	0.32	—	—	—
HSI (%)	5.03 ± 0.08	5.03 ± 0.07	5.11 ± 0.03	5.07 ± 0.06	5.14 ± 0.05	5.18 ± 0.06	0.42	—	—	—
FI (% day^−1^)	4.19 ± 0.09	4.25 ± 0.16	4.16 ± 0.05	4.12 ± 0.05	4.04 ± 0.04	4.16 ± 0.07	0.70	—	—	—
FCR	1.33 ± 0.03	1.35 ± 0.05	1.28 ± 0.04	1.27 ± 0.03	1.24 ± 0.02	1.29 ± 0.02	0.21	—	—	—
PER	1.74 ± 0.03	1.72 ± 0.05	1.82 ± 0.06	1.83 ± 0.04	1.86 ± 0.04	1.80 ± 0.03	0.19	—	—	—
PPV (%)	31.41 ± 0.61	30.75 ± 0.74	32.52 ± 1.05	32.73 ± 0.74	33.40 ± 0.55	32.51 ± 0.66	0.16	—	—	—
Proximate composition (g kg^−1^, dry weight)										
Moisture	740.5 ± 2.2	742.1 ± 1.9	743.9 ± 0.9	745.2 ± 2.0	744.8 ± 1.8	737.5 ± 5.1	0.33	—	—	—
Crude protein	180.6 ± 1.7	179.3 ± 1.9	179.0 ± 1.1	179.5 ± 1.3	179.5 ± 1.5	181.1 ± 1.8	0.93	—	—	—
Crude lipid	20.9 ± 1.2^a^	19.5 ± 0.9^a^	16.1 ± 0.7^b^	16.5 ± 1.2^b^	16.7 ± 0.4^b^	16.6 ± 0.9^b^	<0.001	0.14	0.09	0.23
Ash	36.6 ± 1.4	39.2 ± 0.7	39.9 ± 1.2	38.7 ± 1.1	40.2 ± 0.6	38.2 ± 1.3	0.25	—	—	—

Abbreviations: CF, condition factor; Cu, cubic; FBW, final body weight; FCR, feed conversion ratio; FI, feed intake; HSI, hepatosomatic index; Ln, linear; PER, protein efficiency ratio; PPV, protein productive value; Qd, quadratic; SGR, specific growth rate; SR, survival rate; WGR, weight gain rate.

^1^When the data were not uniform, the Kruskal–Wallis test was used. Otherwise, significant effects among groups were evaluated using a one-way ANOVA. There are no discernible variations between values (mean ± S.E.M., *n* = 6) in a row that have the same superscript letter or none at all (*p* > 0.05). The table displays the specific *p*-value.

^2^Linear, quadratic, and cubic regression models were evaluated for significance using orthogonal polynomial contrasts. Only data with significance are analyzed in the table.

**Table 3:** Effect of dietary bile acids levels on hemolymph metabolism parameters.

Parameters	BA0	BA1	BA2	BA3	BA4	BA5	*p*-Value^1^	Pr > *F*^2^		
								Ln	Qd	Cu
GLU (mmol L^−1^)	9.58 ± 1.13	9.40 ± 1.09	9.59 ± 0.85	8.59 ± 0.75	7.33 ± 0.42	8.50 ± 0.68	0.42	—	—	—
TC (mmol L^−1^)	2.76 ± 0.16	2.94 ± 0.11	2.82 ± 0.05	2.92 ± 0.06	2.81 ± 0.11	3.05 ± 0.09	0.40	—	—	—
TG (mmol L^−1^)	3.47 ± 0.16^a^	3.59 ± 0.08^a^	3.35 ± 0.06^a^	2.98 ± 0.14^b^	2.82 ± 0.11^bc^	2.56 ± 0.15^c^	<0.001	<0.001	0.01	0.02
NEFAs (μmol L^−1^)	142.90 ± 16.88^a^	139.50 ± 12.07^a^	114.33 ± 7.19^ab^	120.17 ± 5.38^ab^	94.92 ± 7.43^b^	108.00 ± 9.07^ab^	0.04	0.08	0.24	0.50
HLD-c (mmol L^−1^)	1.90 ± 0.21	2.19 ± 0.22	2.60 ± 0.24	2.52 ± 0.12	2.52 ± 0.08	2.71 ± 0.11	0.10	—	—	—
LDL-c (mmol L^−1^)	1.83 ± 0.03	1.81 ± 0.05	1.81 ± 0.02	1.80 ± 0.03	1.79 ± 0.03	1.76 ± 0.03	0.86	—	—	—

Abbreviations: Cu, cubic; GLU, glucose; HLD-c, high-density lipoprotein cholesterol; LDL-c, low-density lipoprotein cholesterol; Ln, linear; NEFAs, nonesterified fatty acids; Qd, quadratic; TC, total cholesterol; TG, triglycerides.

^1^When the data were not uniform, the Kruskal–Wallis test was used. Otherwise, significant effects among groups were evaluated using a one-way ANOVA. There are no discernible variations between values (mean ± S.E.M., *n* = 6) in a row that have the same superscript letter or none at all (*p* > 0.05). The table displays the specific *p*-value.

^2^Linear, quadratic, and cubic regression models were evaluated for significance using orthogonal polynomial contrasts. Only data with significance are analyzed in the table.

**Table 4:** Effect of dietary bile acids levels on digestive enzyme activities.

Parameters	BA0	BA1	BA2	BA3	BA4	BA5	*p*-Value^1^	Pr > F^2^		
								Ln	Qd	Cu
Stomach										
Trypsin (U mg prot^−1^)	0.83 ± 0.03	0.84 ± 0.03	0.82 ± 0.03	0.84 ± 0.02	0.82 ± 0.02	0.88 ± 0.01	0.60	—	—	—
Lipase (U g prot^−1^)	0.56 ± 0.03	0.55 ± 0.04	0.55 ± 0.05	0.57 ± 0.03	0.60 ± 0.03	0.59 ± 0.05	0.94	—	—	—
Amylase (U mg prot^−1^)	37.10 ± 2.19	35.62 ± 2.47	34.95 ± 3.42	32.28 ± 2.50	34.48 ± 2.49	34.40 ± 2.19	0.86	—	—	—
Hepatopancreas										
Trypsin (U mg prot^−1^)	209.3 ± 5.14^c^	240.7 ± 7.91^ab^	241.8 ± 9.77^ab^	231.1 ± 11.06^bc^	253.3 ± 6.26^ab^	259.6 ± 4.57^a^	<0.001	0.01	0.03	0.07
Lipase (U g prot^−1^)	1.00 ± 0.07^b^	1.10 ± 0.06^b^	1.50 ± 0.06^a^	1.55 ± 0.07^a^	1.64 ± 0.14^a^	1.65 ± 0.13^a^	<0.001	0.01	0.03	0.12
Amylase (U mg prot^−1^)	41.45 ± 4.19	34.52 ± 2.30	42.78 ± 3.45	34.18 ± 3.09	43.37 ± 3.98	43.07 ± 6.26	0.36	—	—	—
Intestine										
Trypsin (U mg prot^−1^)	12.75 ± 0.61	12.29 ± 0.70	14.15 ± 0.83	13.83 ± 0.70	13.94 ± 0.7	14.32 ± 0.49	0.23	—	—	—
Lipase (U g prot^−1^)	1.71 ± 0.19^b^	2.17 ± 0.17^b^	2.16 ± 0.16^b^	2.77 ± 0.11^a^	2.72 ± 0.20^a^	2.87 ± 0.11^a^	<0.001	0.01	0.03	0.13
Amylase (U mg prot^−1^)	6.66 ± 0.91	6.20 ± 0.43	6.56 ± 0.48	7.31 ± 0.75	7.30 ± 0.61	7.00 ± 0.52	0.79	—	—	—

^1^When the data were not uniform, the Kruskal–Wallis test was used. Otherwise, significant effects among groups were evaluated using a one-way ANOVA. There are no discernible variations between values (mean ± S.E.M., *n* = 6) in a row that have the same superscript letter or none at all (*p* > 0.05). The table displays the specific *p*-value.

^2^Linear, quadratic, and cubic regression models were evaluated for significance using orthogonal polynomial contrasts. Only data with significance are analyzed in the table.

## Data Availability

The raw datasets underlying this research originated from the Zhejiang Marine Fisheries Research Institute. The RNA sequencing data have been archived in the NCBI database under BioProject ID: PRJNA1232396. Derived data underlying this study's conclusions can be obtained by contacting Peng Tan at the corresponding institution.

## Nomenclature

### Abbreviations

alloLCA: Allolithocholic acid
BAs: Bile acids
CF: Condition factor
DEGs: Differentially expressed genes
FCR: Feed conversion ratio
FI: Feed intake
GLU: Glucose
GO: Gene Ontology
HDCA: Hyodeoxycholic acid
HDL-c: High-density lipoprotein cholesterol
HMDB: The Human Metabolome Database
HSI: Hepatosomatic index
isoLCA: Isolithocholic acid
KEGG: Kyoto Encyclopedia of Genes and Genomes
LDL-c: Low-density lipoprotein cholesterol
NEFAs: Nonesterified fatty acids
PCA: Principal component analysis
PER: Protein efficiency ratio
PLS-DA: Partial least squares discriminant analysis
PPV: Protein productive value
SGR: Specific growth rate
SR: Survival rate
TC: Total cholesterol
TCDCA: Taurochenodeoxycholic acid sodium salt
TDCA: Taurodeoxycholic acid sodium salt
TG: Triglycerides
WGR: Weight gain rate

### Gene

*abcg2*: Broad substrate specificity ATP-binding cassette transporter
*acot1*: Acyl-coenzyme A thioesterase 1
*acox1*: Acyl-coenzyme A oxidase 1
*acsl*: Long-chain fatty acid-CoA ligase
*agpat1*: 1-Acyl-sn-glycerol-3-phosphate acyltransferase alpha
*angpt4*: Angiopoietin-4
*dip13a*: DCC-interacting protein 13-alpha
*elovl6*: Very long chain fatty acid elongase 6
*fabp1*: Fatty acid binding protein 1
*fabp4*: Fatty acid binding protein 4
*fasn*: Fatty acid synthase
*gba*: Cytosolic beta-glucosidase
*gstk1*: Glutathione S-transferase kappa 1
*lrp1*: Prolow-density lipoprotein receptor-related protein
*mogat1*: 2-Acylglycerol O-acyltransferase 1
*npc2*: NPC intracellular cholesterol transporter 2
*pck*: Phosphoenolpyruvate carboxykinase
*pfkfb2*: 6-Phosphofructo-2-kinase/fructose-2,6-bisphosphatase 2
*plin3*: Perilipin-3
*pnliprp1*: Inactive pancreatic lipase-related protein 1
*pparα*: Peroxisome proliferator-activated receptor alpha
*prss1*: Trypsin-1
*prss2*: Trypsin-2
*scd*: Stearoyl-CoA desaturase
*slc5a1*: Sodium/glucose cotransporter 1
*spla2*: Group 3 secretory phospholipase A2
*srebp1*: Sterol regulatory element-binding protein 1
*ugt*: UDP-galactose translocator.

## Supporting Information

Supporting Information Table S1: Primers used for qRT-PCR. Table S2: The concentrations of 25 BAs/BA salts in control and BA groups (ng mg^−1^).
