# Supplementary material for: Dietary Bile Acids Supplementation on Growth Performance and Metabolism in Pacific White Shrimp (Litopenaeus vannamei)
Source: Aquac Nutr. 2025 Nov 10;2025:1329200. doi: 10.1155/anu/1329200 (PMC12623099; doi:10.1155/anu/1329200)
Supplement: Supporting Information — Table S1: Primers used for qRT-PCR. Table S2: The concentrations of 25 BAs/BA salts in control and BA groups (ng mg−1). [file 1329200.f1.docx]

**Supplementary Tables**

**Table S1. Primers used for qRT-PCR.**

| **Gene name** | **Gene description** | **Forward primer (5'-3')** | **Reverse primer (5'-3')** | **Product size (bp)** | **PCR efficiency (%)** |
| --- | --- | --- | --- | --- | --- |
| ***b-actin*** | *β-actin* | ACTGGATGGAAGGTGGACAG | TCGCCAACAATGTGCTTTCT | 221 | 100 |
| ***bat1*** | *b(0,+)-type amino acid transporter 1* | TTTCCTCTTCGCGTGGGTAT | CAGCAGGCGATGATCTTGAC | 150 | 101 |
| ***cpa2*** | *carboxypeptidase A2* | GCTGCCGTATGAGCCTAATG | CAGCAGGTCCGTGATATCCT | 220 | 98 |
| ***ef-1a*** | *elongation factor 1-alpha* | AACGTGTCGGTGAAGGATCT | CGATCTTGGTCAGCAGTTCG | 199 | 101 |
| ***elovl6*** | *very long chain fatty acid elongase 6* | TCAAGGCTCTCAAGTTCCGT | CGACACCAACCTGCTTATCG | 249 | 97 |
| ***fabp1*** | *fatty acid binding protein 1* | CGTCACCATCAAGACCGTTG | TCTGGATGAGGTTGCTGCC | 203 | 100 |
| ***fabp3*** | *fatty acid binding protein 3* | TGGAAGGAACGTTCGAACAC | CCTGGTCCTCGTCTGCTAAA | 186 | 99 |
| ***fasn*** | *fatty acid synthase* | ACGGACAAGGAGTGGATGTT | CATCAAAGAGCGAGTCCAGC | 195 | 100 |
| ***fatp4*** | *fatty acid binding protein 4* | CTTCCCGTGTTTGTGTCAGG | CAGCTAAGACACCCCTGGAA | 211 | 97 |
| ***mogat1*** | *2-acylglycerol O-acyltransferase 1* | TGGAGCCTCACTAGTACCCA | GGGGCTCCAACCACTGTATA | 213 | 98 |
| ***pept1*** | *solute carrier family 15 member 1* | GCAGACACTTTCCTTGGACG | GATACCTCCAGTTCCCAGGG | 174 | 100 |
| ***pfkfb2*** | *6-phosphofructo-2-kinase* | TCCATGTCCACAAGCCTTCT | CTTGTACATGTGGGTGGCAC | 222 | 101 |
| ***plin3*** | *perilipin-3* | TATGTGGGAACTGCTTTGCG | CTTTGGCATCTCATCCGTCG | 204 | 99 |
| ***prss1*** | *trypsin-1* | CTCAGTCTGGAAGGTCGAGG | CTTTCACCACCACCTTGTCG | 194 | 100 |
| ***prss2*** | *trypsin-1* | CAACGAGATCTGTGGCATCC | GCGGCAGTGATGATCCAATT | 183 | 100 |
| ***scd*** | *stearoyl-CoA desaturase* | GTGAAACCACCACGATGCTT | GACTCGAGAAGGGAACAGCT | 189 | 99 |
| ***slc5a12*** | *sodium-coupled monocarboxylate transporter 2* | TAGACCCGTATCAGCGACAC | ATGGGGTCGCACTTGTAGAA | 232 | 100 |
| ***srebp1*** | *sterol regulatory element-binding protein 1* | TGCTGGGGAACAATGGGTAT | AAGTTTGCGGGACATTGACC | 186 | 102 |

**Table S2.** **The concentrations of 25 BAs/BA salts in control and BA groups (ng/mg).**

| Bile Acid | Abbreviation | Source category | Structural category | CON | BA |
| --- | --- | --- | --- | --- | --- |
| Taurodeoxycholic acid sodium salt | TDCA | Secondary | Conjugated BAs | 62.21 | 45.63 |
| Taurolithocholic acid sodium salt | TLCA | Secondary | Conjugated BAs | 30.03 | 19.01 |
| Isolithocholic acid | isoLCA | Secondary | Unconjugated BAs | 240.25 | 170.41 |
| Allolithocholic acid | alloLCA | Secondary | Unconjugated BAs | 283.13 | 283.87 |
| Taurochenodeoxycholic acid sodium salt | TCDCA | Primary | Conjugated BAs | 4247.05 | 2265.34 |
| Glycolithocholic acid | GLCA | Secondary | Conjugated BAs | 4.05 | 4.03 |
| Deoxycholic acid | DCA | Secondary | Unconjugated BAs | 4.21 | 7.44 |
| Cholic acid | CA | Primary | Unconjugated BAs | 10.10 | 11.92 |
| Hyodeoxycholic acid | HDCA | Secondary | Unconjugated BAs | 294.41 | 318.79 |
| Chenodeoxycholic acid | CDCA | Primary | Unconjugated BAs | 44.24 | 59.62 |
| 23-Nordeoxycholic acid | 23norDCA | Secondary | Unconjugated BAs | 0.00 | 0.00 |
| 3-dehydrocholic acid | 3-DHCA | Secondary | Unconjugated BAs | 0.00 | 0.00 |
| Glycodeoxycholic acid | GDCA | Secondary | Conjugated BAs | 0.00 | 0.00 |
| Tauroursodeoxycholic acid Dihydrate | TUDCA | Secondary | Conjugated BAs | 0.00 | 0.00 |
| Alpha-Muricholic acid | α-MCA | Primary | Unconjugated BAs | 0.00 | 0.00 |
| Glycohyocholic acid sodium salt | GHCA | Primary | Conjugated BAs | 0.00 | 0.00 |
| Chenodeoxycholic acid-3-beta-D-glucuronide | CDCA-3Gln | Secondary | Conjugated BAs | 0.00 | 0.00 |
| 3beta-Ursodeoxycholic acid | βUDCA | Secondary | Unconjugated BAs | 0.00 | 0.00 |
| Glycoursodeoxycholic acid | GUDCA | Secondary | Conjugated BAs | 0.00 | 0.00 |
| lithocholic acid | LCA | Secondary | Unconjugated BAs | 0.00 | 18.89 |
| 23-Norcholic acid | NorCA | - | Unconjugated BAs | 0.00 | 0.00 |
| Tauro-alpha-Muricholic acid sodium salt | T-α-MCA | Primary | Conjugated BAs | 0.00 | 55.40 |
| Taurohyocholic acid sodium salt | THCA | Primary | Conjugated BAs | 0.00 | 0.00 |
| Glycocholic acid hydrate | GCA | Primary | Conjugated BAs | 0.35 | 0.29 |
| 7-Ketolithocholic acid | 7-ketoLCA | Secondary | Unconjugated BAs | 0.00 | 0.00 |
